# Wogonin induces retinal neuron-like differentiation of bone marrow stem cells by inhibiting Notch-1 signaling

**DOI:** 10.18632/oncotarget.16085

**Published:** 2017-03-10

**Authors:** Qinmeng Shu, Hong Zhuang, Jiawen Fan, Xin Wang, Gezhi Xu

**Affiliations:** ^1^ Department of Ophthalmology and Vision Sciences, Eye and ENT Hospital, Shanghai Medical College, Fudan University, Shanghai, People's Republic of China; ^2^ Shanghai Key Laboratory of Visual Impairment and Restoration, Shanghai, People's Republic of China

**Keywords:** wogonin, bone marrow stem cells, Notch-1, retinal degenerative diseases, differentiation

## Abstract

Age-related macular degeneration and retinitis pigmentosa are major causes of irreversible vision loss in the elderly and, despite sustained efforts, current treatments are largely ineffective. Wogonin is a bioactive plant flavonoid possessing a range of beneficial properties, including neuroprotective effects. We investigated the ability of wogonin to promote retinal neuron-like differentiation of bone marrow stem cells (BMSCs) and assessed the involvement of Notch-1 signaling in this process. Cultured mouse BMSCs were left untreated or exposed to neurotrophic factors in the presence or absence of wogonin, and western blotting, RT-PCR and immunofluorescence were used to identify changes in molecular markers of stemness and neuroretinal differentiation. Proteins in the Notch-1 signaling pathway, a main negative regulator of neurogenesis, were also examined by western blotting. We found that expression of stem cell markers was reduced, while markers of mature retinal neurons, bipolar cells and photoreceptors were increased in wogonin-treated BMSCs. Wogonin also dose-dependently decreased expression of Notch-1 signaling proteins. Moreover, blockade of Notch-1 both mimicked and enhanced the effect of wogonin to facilitate BMSC differentiation into retinal neuron-like cells. Wogonin thus appears to promote retinal neuron-like differentiation of BMSCs by antagonizing the inhibitory actions of Notch-1 signaling on neurogenesis and may be useful in the treatment of retinal degenerative diseases.

## INTRODUCTION

Retinal degenerative diseases, such as age-related macular degeneration and retinitis pigmentosa, are the major cause of irreversible vision loss in the elderly [1]. They are characterized by irrevocable retinal pigment epithelium (RPE) loss followed by degeneration of adjacent photoreceptors [2, 3]. Owing to the tremendous neuronal regeneration impediment in higher mammals, there are currently no broadly effective treatments for retinal degeneration disorders. Stem cell-derived tissue regeneration therapy provided proof-of-promise that replacement of dysfunctional retinal neurons and RPE can rescue vision in patients with severe retinal degenerative diseases [4, 5]. Previously, studies had demonstrated that the neurotrophic secretome of bone marrow stem cells (BMSCs) prevented degenerating retina from deteriorating and that differentiated BMSCs improved the function of the damaged retina by replenishing ineffective neurons [6–8]. However, the limited neural differentiation efficacy of BMSCs hinders these cells from regenerating functional retinal elements [6]. Therefore, finding an optimized method for efficiently deriving RPE cells and retinal neurons from stem cells is imperative.

Wogonin (5,7-dihydroxy-8-methoxyflavone), a flavonoid compound that originates from the roots of Scutellaria baicalensis Georgi [9], has been reported to have a variety of bioactive effects including antioxidant, antiinflammatory, and anticancer activities [10–12]. Moreover, neuroprotective effects have also been ascribed to wogonin in different nerve injury models. For instance, wogonin protected rat dorsal root ganglion neurons from endoplasmic reticulum stress-induced apoptosis, and ameliorated LPS-induced inflammatory responses by inhibiting TLR4 signaling [13, 14]. In addition, wogonin significantly improved histological and functional outcomes in mice cortical neurons after experimental traumatic brain injury [15]. Furthermore, Lim and colleagues found that wogonin, without any additional co-factors, efficiently promoted neurite outgrowth in primary cortical neural precursor cells (NPCs) cultures from neonatal rat cerebra [16]. Zhang et al. studied the mechanisms of neurite formation promoted by wogonin and speculated that it may be mediated by activation of cell cycle reentry signals, including PKCδ and p21, therefore promoting neurite outgrowth and transmission of neurotrophic factors for neuronal survival [17, 18]. Thus, available data indicate that wogonin facilitates the maturation of NPCs to form a functioning neurotrophic network, and this may be the underlying basis of its neuroprotective effects.

As the influence of wogonin on the neurogenic potential of BMSCs remains undefined, in this study we performed molecular marker analyses to test the hypothesis that wogonin enhances neural differentiation in cultured mice BMSCs. In addition, we investigated the underlying mechanism and found that wogonin's neural induction properties are related to Notch-1 signaling inhibition in these cells.

## RESULTS

### Effect of wogonin on BMSCs viability

**Figure 1:**
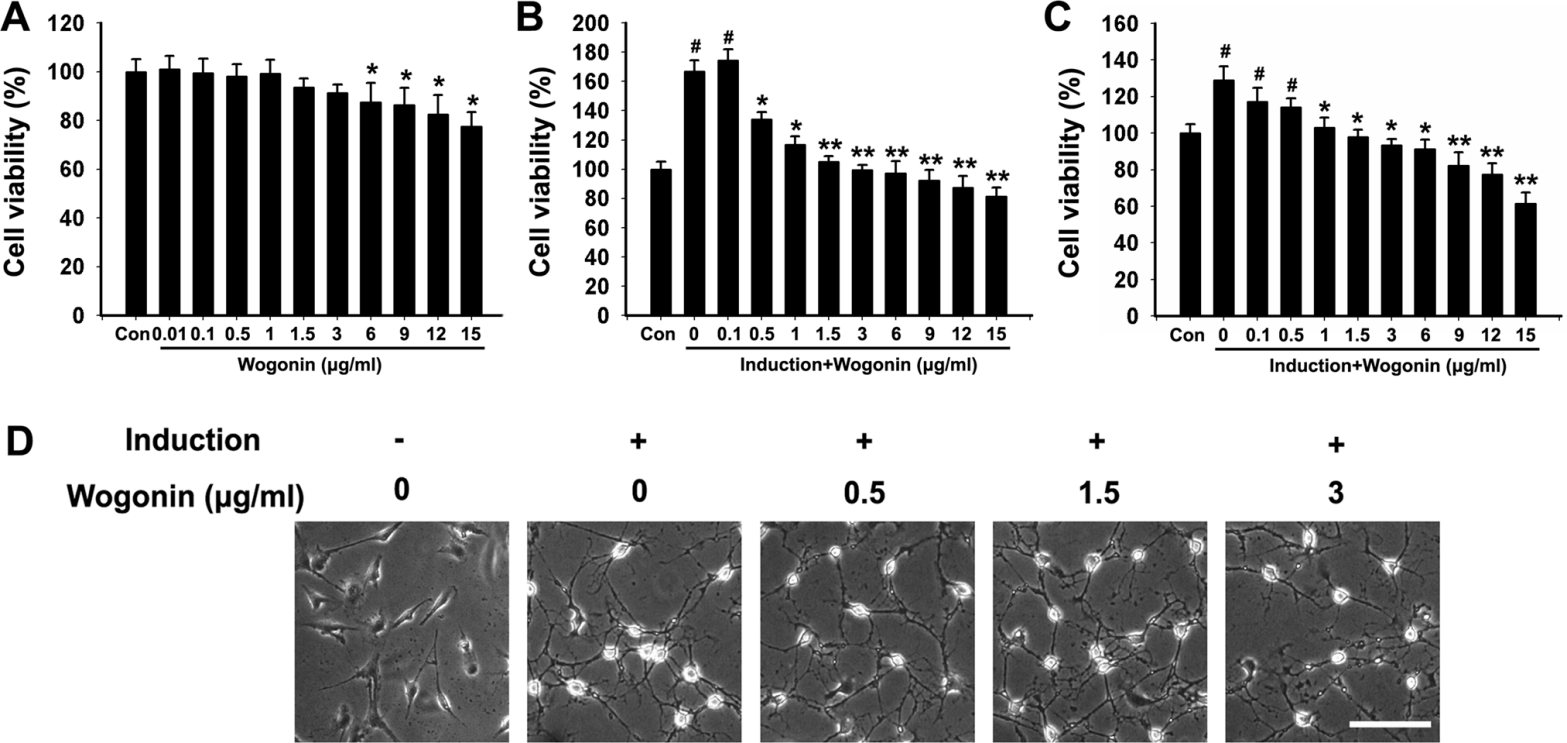
Cell viability and morphological changes in BMSCs after neural induction and wogonin treatment (**A**) Effects of wogonin on BMSCs viability. Cell activity decreased by 18%-29% in BMSCs treated with 6-15 μg/ml of wogonin (*p < 0.05). (**B**) BMSCs viability after 24h of neural induction (#*p* < 0.05, neural induction and wogonin versus control group; **p < 0.01, *p < 0.05, wogonin versus standard neural induction group). (**C**) BMSCs viability changes after 48h of induction. (**D**) Morphological changes in BMSCs 14 days after induction. Scale bar = 25 μm.

Mice BMSCs cultures were adherent and displayed a spindle-like shape (Figure 1D). As shown in Figure 1A, no apparent differences in cell viability existed between control and wogonin-treated cells at wogonin concentration below 3μg/ml. Cell viability tended to gradually decline in a concentration-dependent manner when treated with ≥ 6 μg/ml of wogonin (*p < 0.05). Thus, a decrease in cell growth activity of 18%–29% corresponded to wogonin concentrations of 6–15 μg/ml. We therefore set a ceiling concentration of 3 μg/ml for subsequent experiments.

BMSCs cultures were established as follows: 1) Control (no drugs or factors); 2) Standard Neural Induction (containing neuronal differentiation factors; see Mat. & Methods); and 3) Wogonin (Neural Induction treatment plus wogonin). As shown in Figure 1B, 24 h after induction BMSCs showed markedly increased growth activity (#*p* < 0.05), which was reduced instead in the wogonin group (*p < 0.05, compared to cells induced without wogonin). The restricting effect of wogonin on cell viability seemed obvious at its lowest concentration (0.5 μg/ml; *p < 0.05), and was more significant above 1 μg/ml (**p < 0.01). Figure 1C shows that compared to these changes, cell growth changes in BMSCs induced without wogonin showed, after 48h of culture, the same tendency but a milder slope.

### Wogonin prompts faster depletion of pluripotency in BMSCs

**Figure 2:**
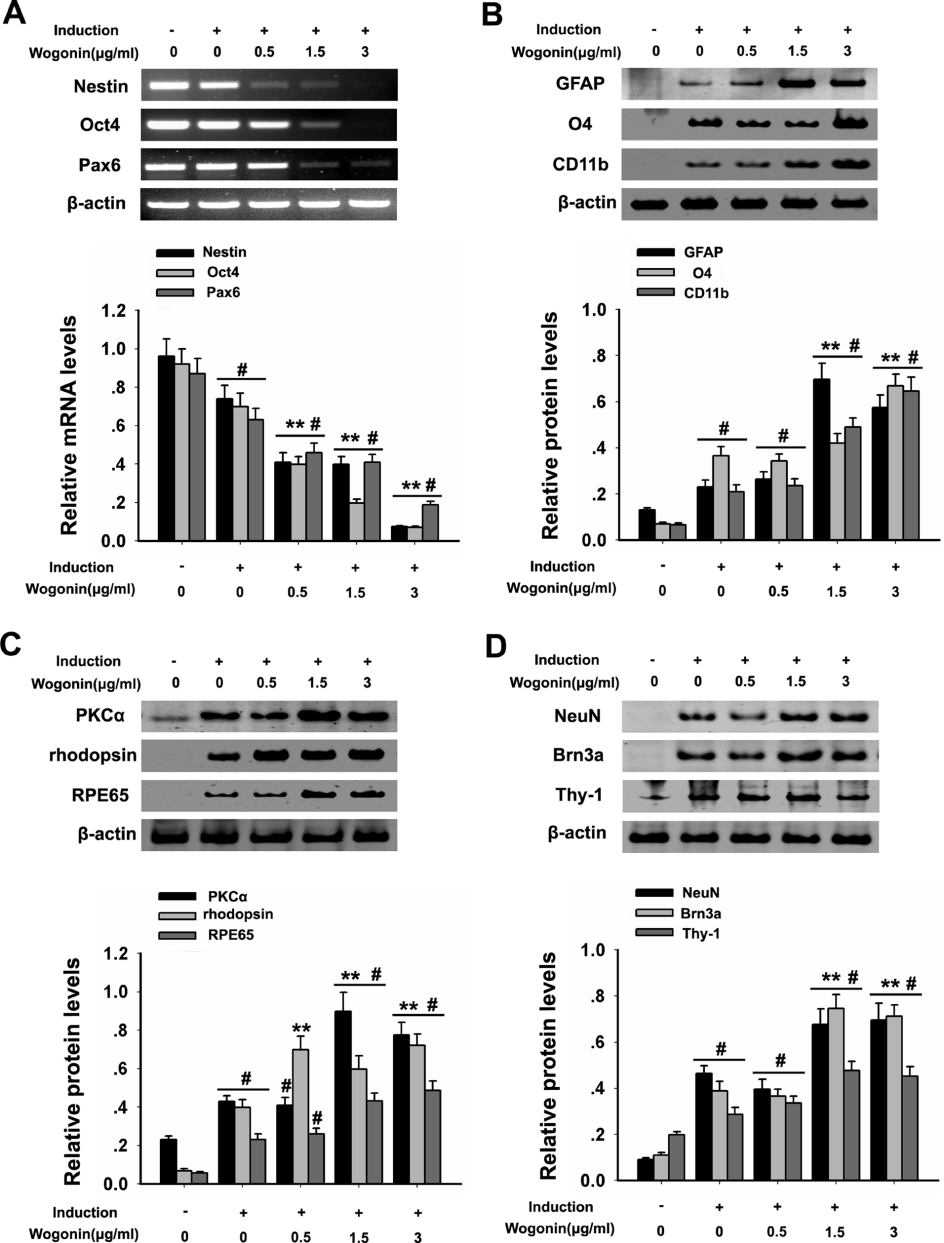
Wogonin enhances neural differentiation of BMSCs BMSCs were harvested at day 14 after induction and subjected to RT-PCR and western blot analyses. (**A**) mRNA levels of nestin, Pax6 and Oct4 examined by RT-PCR. β-actin was used to ensure equal loading. (**B**). Protein analysis of glial markers (O4, CD11b and GFAP) by western blot. (**C**) Protein analysis of PKCα, rhodopsin, and RPE65 by western blot. (**D**) Protein analysis of NeuN, Brn3a, and Thy-1 by western blot. β-actin was used to ensure equal loading of all groups. Data are shown as mean ± SEM (*n* = 6 per group; #*p* < 0.05 neural induction versus control group, ^*^p < 0.01 wogonin versus neural induction group).

**Figure 3:**
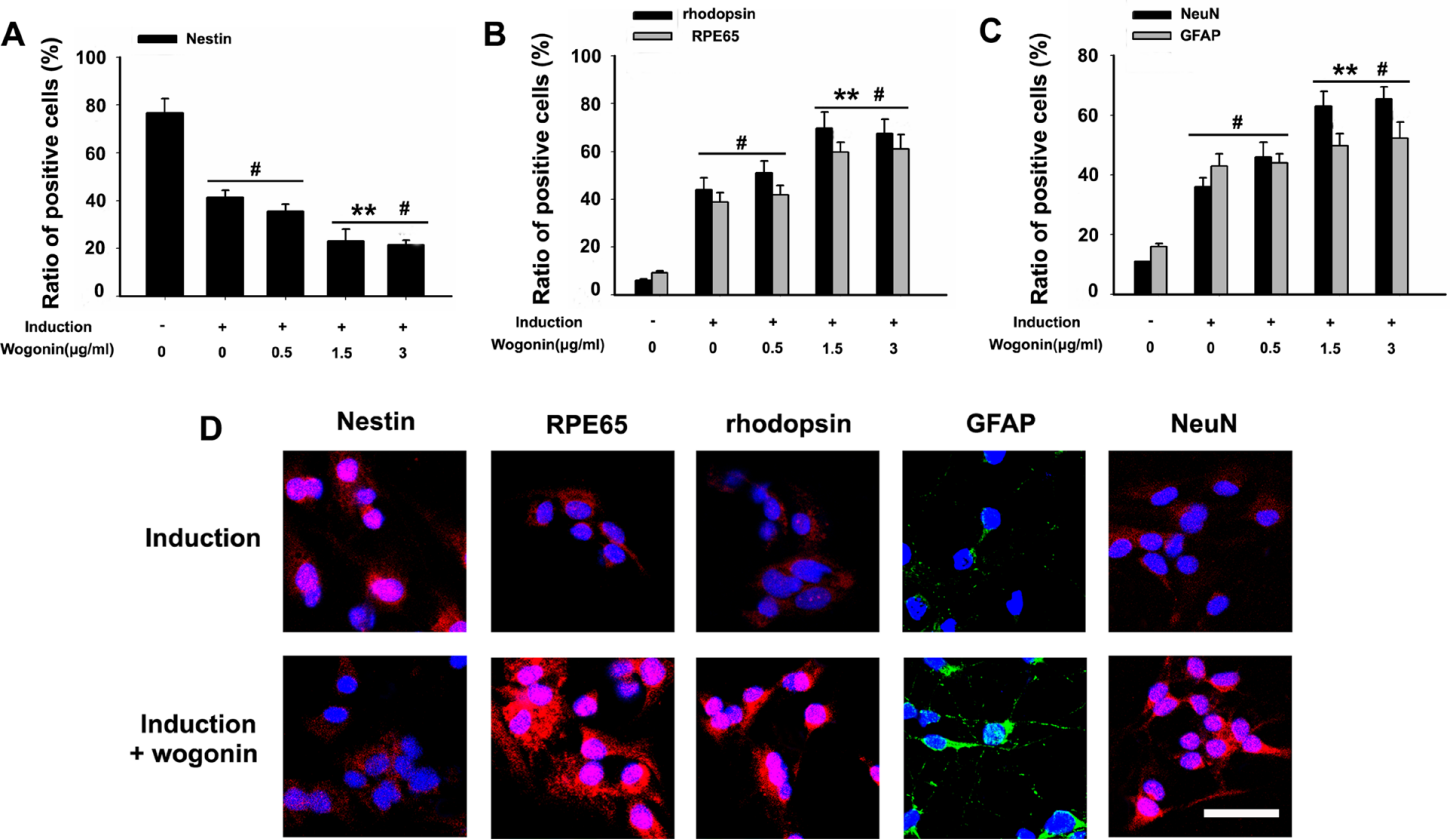
Effect of wogonin on the expression of stemness and neuroretinal markers BMSCs were harvested 14 days after induction and subjected to immunostaining. (**A–C**) Analysis of the effects of wogonin on nestin, rhodopsin, RPE65, GFAP, and NeuN expression by counting the number of positive cells over the total BMSC count. Scale bar = 25 μm (**D**). Neurally induced BMSCs grown with or without wogonin were stained with antibodies against nestin, rhodopsin, RPE65, GFAP and NeuN. Data are shown as mean ± SEM (*n* = 6 per group, #*p* < 0.05, standard neural induction versus control group; ^*^p < 0.01, wogonin versus standard induction group).

To investigate the differentiation potential of BMSCs, RT-PCR was used to examine stemness markers 14 days after neural induction. Results showed that, compared with the control group, the expression of Oct4, Pax6 and nestin were significantly decreased (^#^*p* < 0.05; Figure 2A), while a more drastic decrease occurred in BMSCs in the wogonin group, compared with the standard neural induction group (**p < 0.01; Figure 2A). In addition, immunofluorescence showed that the proportion of nestin-positive BMSCs was significantly reduced in the wogonin group (Figure 3D).

### Wogonin enhances neural differentiation of BMSCs

After 14 days of induction, BMSCs from both wogonin and standard induction groups displayed neuron-like characteristic neurite outgrowth (Figure 1D), and showed a significantly increased expression of GFAP, O4, and CD11b (retinal glia markers), PKCα (bipolar cells’ marker), NeuN, Brn3a, Thy-1 (retinal ganglion cell markers), RPE65 (RPE marker), and rhodopsin (rod photoreceptor marker), compared with cells in the control group (#*p* < 0.05; Figure 2B–2D). Obvious alterations in the expression of several markers were further observed between BMSCs from the wogonin group and the standard induction group. The levels of GFAP, O4, CD11b, PKCα, NeuN, Brn3a, Thy-1, RPE65 and rhodopsin were all significantly higher (increased more than two-fold) in the wogonin group (**p < 0.01; Figure 2B–2D).

To further verify the differences between neurally induced BMSCs with or without concurrent wogonin treatment, immunofluorescent labeling was performed in both groups 14 days after induction. In the standard induction group the positive-to-negative-cell staining ratios for GFAP (43% ± 5.6), NeuN (37% ± 2.9), RPE65 (39% ± 1.7), and rhodopsin (44% ± 2.3) were lower than those quantified in the wogonin group (52% ± 1.8, 64% ± 3.0, 60% ± 2.5, and 68% ± 4.8, respectively; Figure 3A–3D). These findings were consistent with the results of Western blot analyses described above.

### Wogonin inhibits Notch-1 signaling during BMSCs neural differentiation

**Figure 4:**
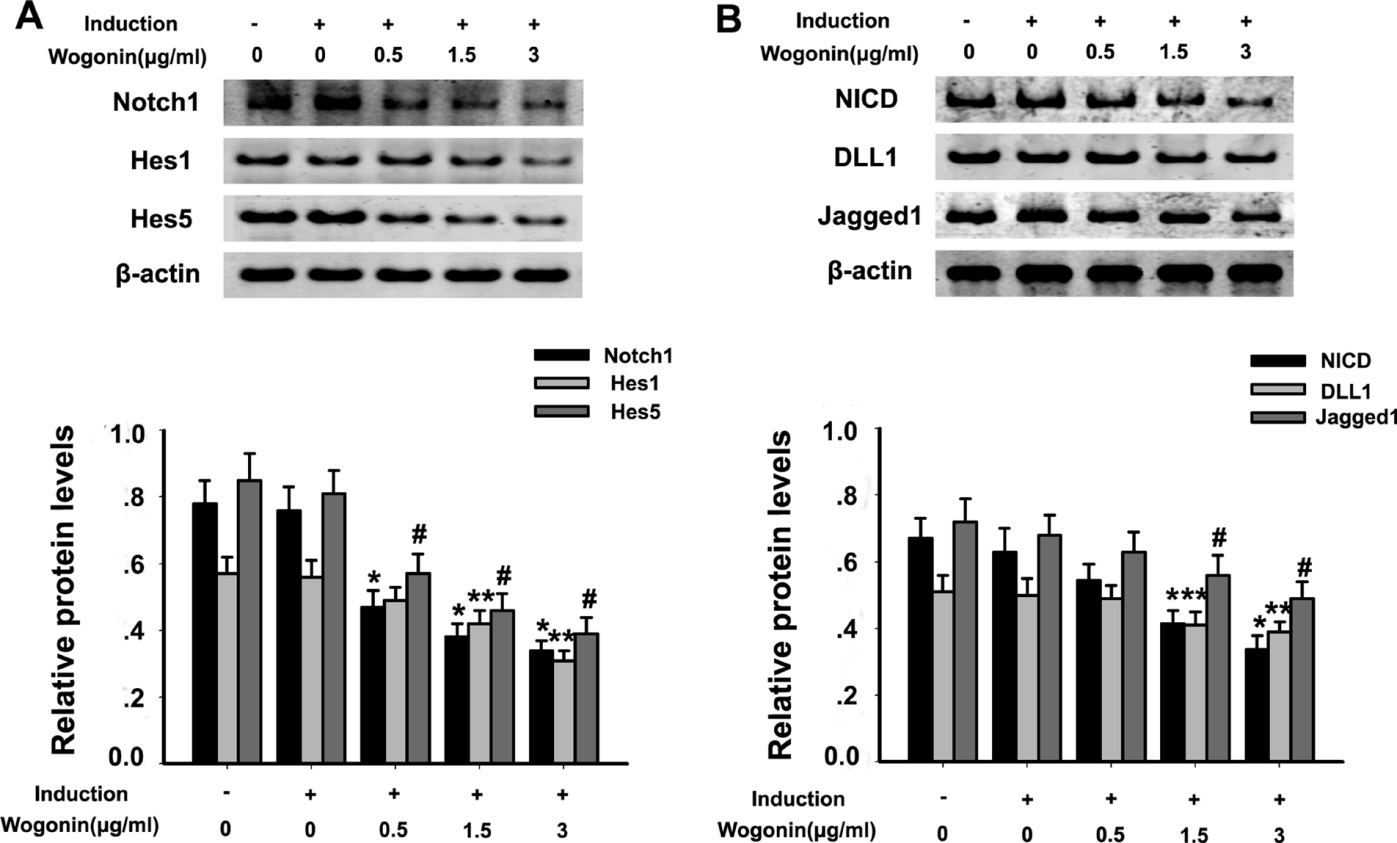
Effect of wogonin on Notch-1 signaling protein expression in BMSCs BMSCs from different experimental groups were treated with or without the Notch-1 inhibitor DAPT and subjected to western blot analysis. (**A**) Protein levels of Notch-1, Hes1 and Hes5. (**B**) Protein levels of NICD, Jagged1 and DLL1. Densitometric analysis shows the effects of wogonin on the expression of these proteins. Data are shown as mean ± SEM (*n* = 6 per group, #*p* < 0.05 neural induction versus control group, *p < 0.05, ^*^p < 0.01 wogonin versus induction group).

The Notch-1 pathway controls NPC self-renewal and is a critical regulator of neuronal embryogenesis. To investigate whether wogonin's effects on BMSC neural induction involve changes in Notch-1 signaling, the expression of Notch-1, Notch-1 ligands and their downstream pathway proteins was examined by Western blot in neurally induced cells exposed or not to wogonin. While both conditions led to a decreased expression of Notch-1, NICD, DLL1, Jagged1, Hes1, and Hes5 (#*p* < 0.05; Figure 4A–4B), a further, significant reduction in the expression of these proteins was detected in the wogonin group (**p < 0.01; Figure 4A–4B).

### Wogonin coordinates with Notch-1 inhibition in promoting neural differentiation of BMSCs

**Figure 5:**
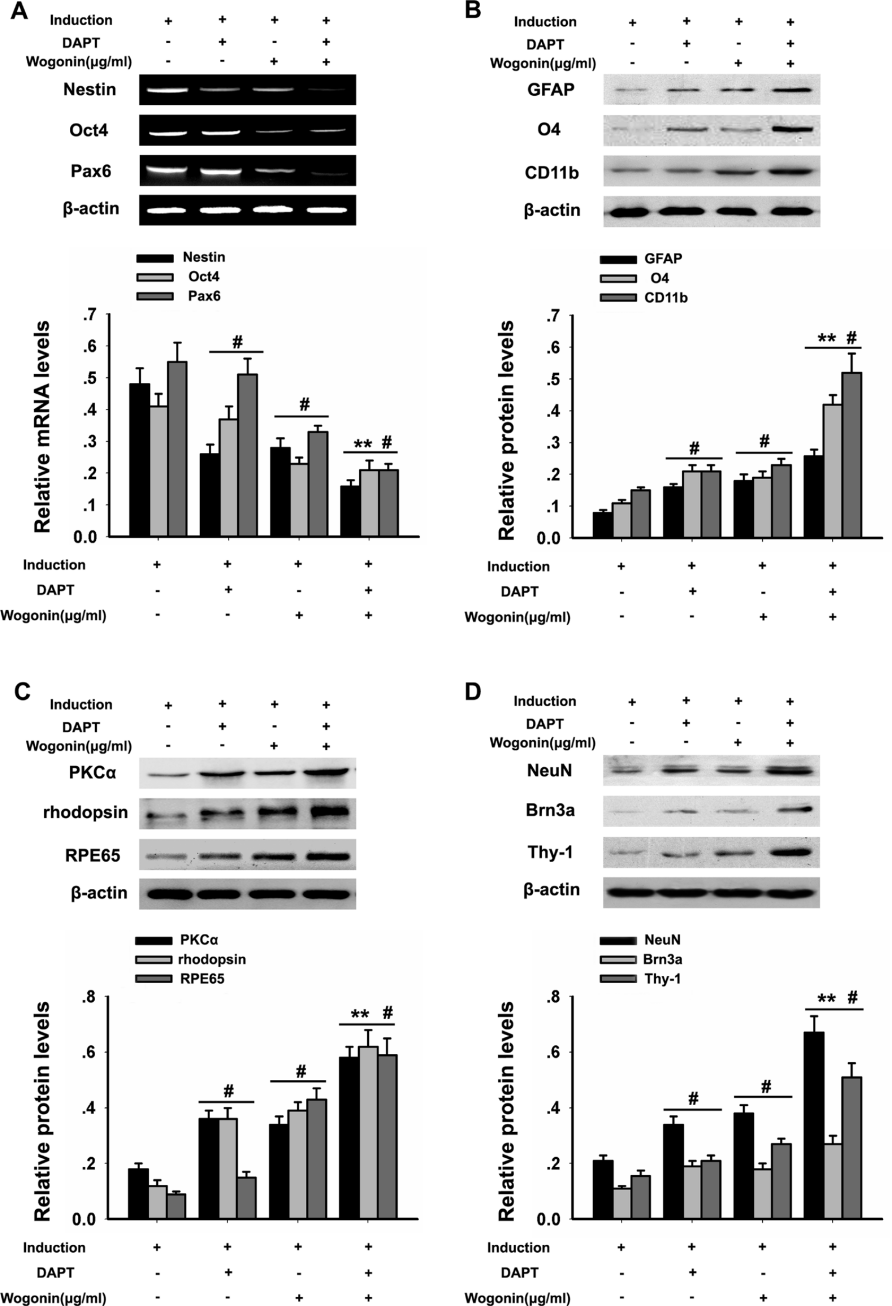
Effects of wogonin and DAPT on neural differentiation of BMSCs BMSCs from the different experimental groups were treated with or without DAPT/wogonin combination and subjected to RT-PCR and western blotting analyses. (**A**) mRNA levels of Nestin, Pax6 and Oct4 examined by RT-PCR. Densitometric analysis of the effect of wogonin on Nestin, Pax6 and Oct4 protein expression (**B**). Protein levels of glial markers (O4, CD11b and GFAP) examined by western blot. (**C**) PKCα, rhodopsin, and RPE65 levels assessed by western blot. (**D**) NeuN, Brn3a, and Thy-1 protein expression assessed by western blot. β-actin was used to ensure equal loading in all groups. Data are shown as mean ± SEM (*n* = 6 per group, #*p* < 0.05, DAPT or wogonin versus neural induction group, ^*^p < 0.01, wogonin-plus-DAPT versus DAPT group).

**Figure 6:**
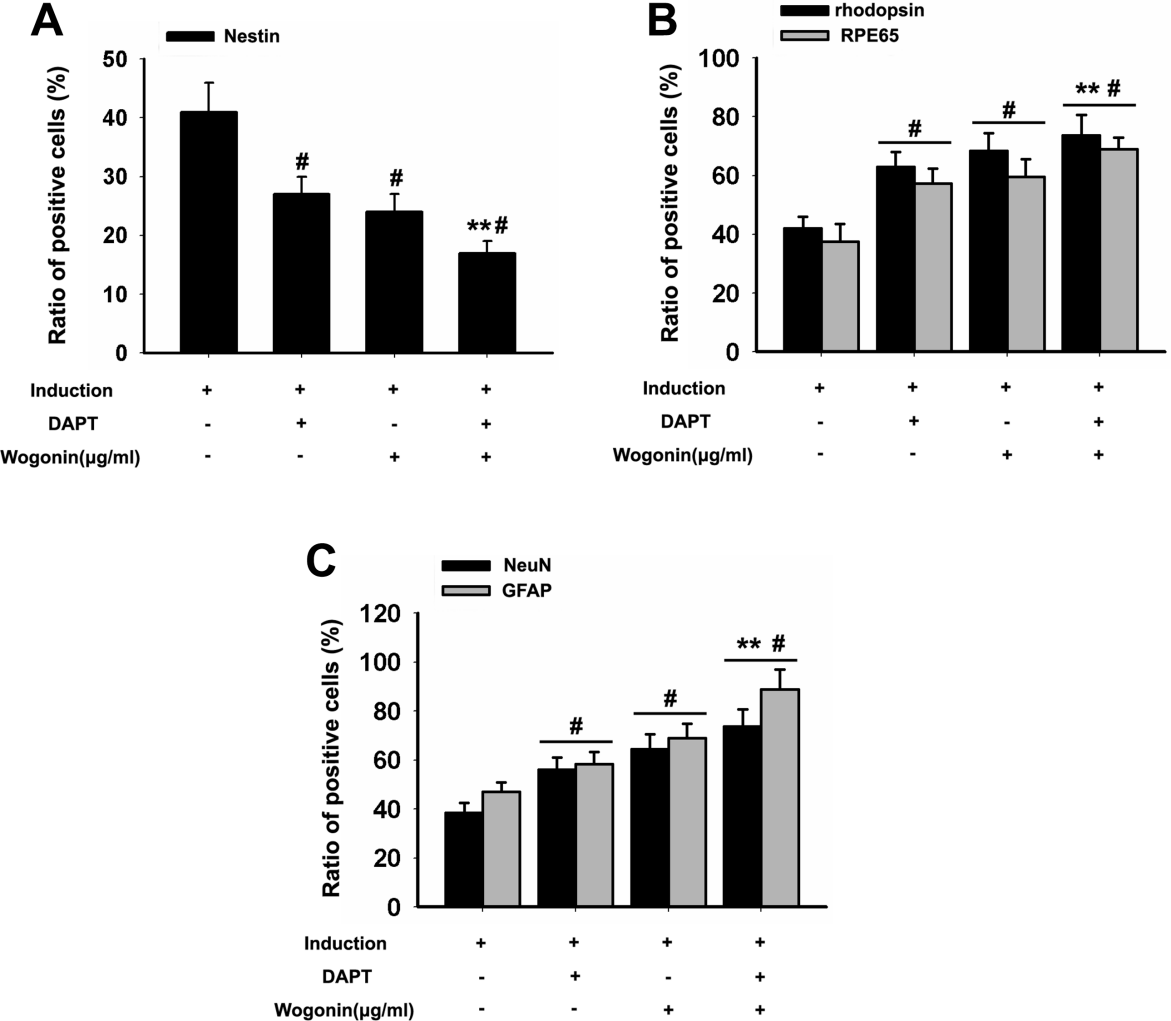
Effect of wogonin and DAPT on the expression of stemness and neuroretinal markers BMSCs were harvested 14 days after induction and subjected to immunostaining. (**A–C**) Densitometric analysis of the effects of wogonin on the expression of stemness and neuron-like markers was assessed by counting the number of nestin-, rhodopsin-, RPE65- GFAP-, and NeuN-positive cells over the total BMSC count. Data are shown as mean ± SEM (*n* = 6 per group, #*p* < 0.05, DAPT or wogonin versus neural induction group; ^*^p < 0.01, wogonin-plus-DAPT versus DAPT group).

To better examine the relationship between wogonin, Notch-1 signaling and the neurogenic potential of BMSCs, we used RT-PCR and Western blot to evaluate changes in Notch-1 pathway proteins and neuronal marker in response to Notch-1 inhibition. BMSCs were cultured in neural induction medium in the presence or absence of the Notch-1 inhibitor DAPT for 14 days. Western blots showed that DAPT decreased significantly the expression of Notch-1, NICD, DLL1, Jagged1, Hes1, and Hes5 (*p < 0.05, **p < 0.01; Figure 4A–4B). Addition of wogonin to DAPT-treated, induced cells maximally reduced the mRNA levels of the stemness markers nestin, Oct4, and Pax6 (**p < 0.05; Figure 5A) and increased the levels of several neuroglial and retinal cells’ proteins such as GFAP, O4, CD11b, PKCα, NeuN, Brn3a, Thy-1, RPE6,5 and rhodopsin (*p < 0.05, **p < 0.01; Figure 5B–5C). The downregulation of nestin, and the enhanced expression of neuroglial/retinal cells’ markers in DAPT- and wogonin-treated BMSCs were further validated using immunofluorescence (#*p* < 0.05, **p < 0.01; Figure 6A–6C).

## DISCUSSION

The bioactive plant flavonoid wogonin has been addressed for its ability to induce neural differentiation in NPCs. In line with previous studies, we validated here a role for wogonin as an inducer of neuron-like cell differentiation in mice BMSCs. RT-PCR, western blot, and immunofluorescence analyses of neuroglial and retinal cell differentiation markers suggested that BMSCs treated with wogonin adopted a phenotype that resembled that of retinal neurons.

Changes in BMSC pluripotency were first examined by RT-PCR. Results showed that relative mRNA levels of Oct4, nestin, and Pax6, characteristically expressed in neural primitive/progenitor pluripotent cells of the developing vertebrate brain [19–21], were significantly lower in cells induced with neurotrophic factors in the presence of wogonin. In accordance with the results of RT-PCR, immunofluorescence showed that the number of nestin-positive BMSCs were much lower in neurally induced cells treated with wogonin. These observations indicate that wogonin decreased stemness properties and accelerated the process of differentiation of BMSCs.

The differentiation efficiency of wogonin was further evaluated by analyzing retinal cells’ markers through western blotting and immunofluorescence. Consistently, both techniques showed that the protein levels of retinal neuron markers such as RPE65, rhodopsin, NeuN and GFAP were significantly elevated in wogonin-treated BMSCs, compared with cells induced without wogonin. Combined with morphological observations (i.e. wogonin-induced neurite outgrowth), these results demonstrate that when combined with neurotrophic factors, wogonin prompts BMSCs to differentiate into generic retinal-like neurons with greater efficiency. Given the fact that wogonin, when applied alone, could not initiate the differentiation of BMSCs, we speculate that wogonin might affect certain intrinsic, neurogenic signaling pathways in BMSCs to enhance their response to neural induction factors.

Differentiated, neuron-like BMSCs expressed a relatively wide spectrum of retinal neuron markers, a phenomenon that shared some similarities with the early stages of retinogenesis [22, 23]. During retinal development, retinal neurons arise in a conserved, temporal sequence from multipotent, cycling retinal progenitor cells (RPCs) [24]. RPCs are initially pluripotent and are the fundamental cells giving rise to all types of retinal neurons [25]. Prior to this phase, the evolutionarily conserved Notch-1 signaling contributes to RPC proliferation and forestalling of retinal neurogenesis in order to maintain an adequate progenitor cell pool [26, 27]. Therefore, we investigated whether Notch-1 signaling plays a similar role in the neural differentiation of BMSCs. Predictably, the expression of Notch signaling proteins, namely the receptor Notch-1, the ligands Jagged1, Deltalike1 (DLL1), and the downstream effectors Hes1 and Hes5, was significantly reduced as BMSC differentiation progressed. Accordingly, an enhanced neural differentiation efficiency, concomitant with a significantly decreased expression of Notch-1 pathway proteins, was observed in induced BMSCs treated with DAPT, a Notch-1 inhibitor. Interestingly, BMSCs cultured with wogonin exhibited a more rapid decline in the expression of Notch-1 cascade proteins, indicating that the inhibitory effect of Notch-1 signaling on neurogenesis was further lessened. These data suggest that after induction, Notch-1 signaling activity gradually fades, switching BMSCs from a relatively static state to a neurogenic state. Moreover, these results imply that wogonin may quicken the process of neuronal differentiation through inhibition of Notch-1 signaling. This evidence further indicates that the effects of DAPT and wogonin are superimposed and may share similar molecular mechanisms.

Notch-1 determines specific neurogenic cells fates through lateral inhibition [28, 29]. To implement lateral inhibition, cells fated to differentiate exhibit a pro-neurogenic pattern and transactivate Notch-1 signaling in adjacent cells through increased production of (Notch-1)-Ligands (DLL1, Jagged1) [30]. This reciprocal regulation gives rise to neurons by inhibiting their intracellular Notch-1 signaling while simultaneously repressing neuronal differentiation by activating Notch receptors in neighboring cells [31–33]. In contrast with the expression patterns observed during retinogenesis, the down-regulation of both Notch-1 receptor and the Notch ligands DLL1 and Jagged1 suggested that the crosstalk bridged by Notch-1 between differentiating BMSCs was abolished, and rather than lateral inhibition the cells showed a synchronized pattern of differentiation.

Hes1 and Hes5 are basic helix-loop-helix (HLH) factors [34, 35]. The HLH domain endows Hes genes with DNA binding function to capture the promoter or regulatory sequences of target genes in order to enrich transcriptional co-repressors and implement direct repression [36–38]. Apart from this function, Hes1 expression cyclically oscillates and is inversely correlated with DLL1 expression, suggesting a role for the Hes gene family in lateral inhibition [39]. In the present study, we observed that wogonin not only suppressed membrane proteins involved in Notch-1 signaling, but also interfered with the nuclear translocation of the Notch-1 intracellular domain (NICD), therefore preventing the transcription of the neurogenesis suppressors Hes1 and Hes5, and congruently permitting neuron-specific gene expression and neuronal differentiation. The simultaneous decline in both Hes1 and Hes5 protein expression in BMSCs treated with wogonin suggests that wogonin may lead to a strengthened suppression of Hes genes, abolishing the inhibition that Hes proteins exert on neurogenesis.

In summary, our study shows that neurally induced BMSCs exposed to wogonin differentiated into retinal neuron-like cells that exhibited a generic spectrum of retinal neuron markers influenced by Notch-1 signaling, and shared some features of RPCs during the early stages of retinal development but in a distinct, fine-tuned way. Our investigation suggests that wogonin enhances in BMSCs a neurogenic state induced by classic neurotrophic factors by suppressing Hes gene expression and in the absence of lateral inhibition, implying a generally suppressive role of wogonin on Notch-1 signaling cascade. In conclusion, the greater efficiency of wogonin in eliciting retinal neuron-like differentiation of BMSCs holds promise for translating this knowledge into clinical practice, and may offer a potential new therapy to treat retinal degenerative diseases.

## MATERIALS AND METHODS

### Animals

The present investigation conformed to the Law of the People's Republic of China on the Protection of Wildlife, and the protocol was approved by the Animal Ethics Committee of the Eye and ENT Hospital of Fudan University. All the experimental procedures were carried out in accordance with the ARVO Statement for the Use of Animals in Ophthalmic and Vision Research. Six- to eight-week-old male Balb/c mice with a body weight of 20 ± 2 g were used in this study. Mice were bred on a 12-hour light/12-hour dark cycle and were given free access to food and water in a pathogen-free environment.

### Preparation of BMSCs

To generate BMSCs, bone marrow mononuclear cells were isolated from the femurs and tibiae of at least five mice to minimize cell variability. The bone marrow was flushed out from the bones using a syringe containing 5 ml phosphate-buffered saline (PBS) with 100 U/ml heparin, and centrifuged for 8 min. Monolayered cells were suspended in 10 ml culture medium with 10% fetal bovine serum (DMEM; Gibco, OK, USA) and seeded in a sterilized flask (Corning Inc., NY, USA). Cells were cultured in a standard humidified environment at 37°C. The medium was completely replaced and non-adherent cells were removed every 3 days. Approximately 7–10 days after seeding, the adherent cells were digested by using 0.25% trypsin (Sigma, MO, USA) and transferred to sterilized flasks. After 3 to 5 passages, the BMSCs were utilized for further experiments.

### Cell viability assay

Cell viability was examined by using the MTT assay. Spent medium was discarded and 10 μl of MTT solution (5 mg/ml) was added to 100 μl of DMEM for each well. After XX hours, 100 μl of DMSO per well were added and absorbance was examined at 570 nm with a microplate reader (Bio-Rad, CA, USA). Cytotoxicity, represented by growth inhibition, was expressed as percentage of cell viability.

### Experimental groups

BMSCs were divided into three experimental groups: 1) Control: BMSCs without any added co-factors. 2) Standard Neural Induction: To induce neural differentiation, BMSCs (1 × 10^3^) were replated and supplemented with 2% B27, 2% N2 (PAA Laboratories, Germany), 25 ng/ml BDNF, 40 ng/ml NGF, and 25 ng/ml bFGF (R&D Systems, MN, USA). 3) Wogonin: Standard neural induction treatment plus wogonin (ALX-385-033-M005, Alexis, USA) 0.5 μg/ml, 1.5 μg/ml, or 3.0 μg/ml, dissolved in 30% dimethyl sulfoxide (Concentration gradient). To determine the effects of Notch-1 signaling on neural differentiation, BMSCs in each group were grown in the presence or absence of the Notch-specific inhibitor DAPT (10 ng/ml; Sigma, MO, USA). After 14 days of treatment BMSCs were used for further experiments.

### Immunofluorescence

BMSCs were fixed with 4 % paraformaldehyde at days 7 or 14 post-neural induction, blocked in 1 % bovine serum albumin and 0.2% Triton X-100 (Sigma-Aldrich, MO, USA), and incubated at 37°C for 1h with primary antibodies against glial fibrillary acidic protein (GFAP; 1:200; rabbit anti mouse; Santa Cruz, CA, USA), nestin (a neural stem cell marker; 1:200; Sigma, goat anti mouse; MO, USA), neuron-specific nuclear protein (NeuN; retinal ganglion cell marker; 1:200; rabbit anti mouse; Abcam, MA, USA), RPE65 (an RPE marker; 1:200; rabbit anti mouse; Santa Cruz, CA, USA) and rhodopsin (a photoreceptor marker; 1:200; rabbit anti mouse; Abcam, MA, USA). The cells were subsequently incubated at 37°C with fluorescein-conjugated secondary antibodies (Dako, CA, USA), mounted and observed under a fluorescence microscope.

### Western blotting analysis

Total cellular protein was extracted using the ProteoPrep Total Extraction Sample Kit (Sigma-Aldrich, MO, USA) following the manufacturer's instructions. The protein sample purity was tested by a protein assay (Sigma-Aldrich, MO, USA). Fifty milligrams of total protein sample were resolved on SDS-PAGE gels, transferred onto polyvinylidene membranes (Millipore, MA, USA) and blocked with skimmed milk for 1h. Then the samples were incubated overnight at 4°C with primary antibodies against GFAP (1:200; Santa Cruz, rabbit anti mouse; CA, USA), Nestin (1:200; goat anti mouse; Sigma, MO, USA), neuron-specific nuclear protein (NeuN; 1:200; rabbit anti mouse; Abcam, MA, USA), RPE65 (1:200; rabbit anti mouse; Santa Cruz, CA, USA), rhodopsin (1:200; rabbit anti mouse;Abcam, MA, USA), O4 (1:200; rabbit anti mouse; Cell Signaling, MA, USA), Hes5 (1:200; goat anti mouse; Cell Signaling, MA, USA), PKCα (1:500; rabbit anti mouse;Cell Signaling, MA, USA), DLL1 (1:200; rabbit anti mouse; Santa Cruz, CA, USA), CD11b (1:1,000; rabbit anti mouse; Abcam, MA, USA), Notch-1 (1:500; goat anti mouse; R&D, MN, USA), Jagged1 (1:500; goat anti mouse; Cell Signaling, MA, USA), NICD (Notch intracellular domain; 1:500; goat anti mouse; Cell Signaling, MA, USA), Hes1 (1:200; goat anti mouse; Cell Signaling, MA, USA), Brn3a (1:200; goat anti mouse; Cell Signaling, MA, USA), Thy-1 (1:500; rabbit anti mouse; Santa Cruz, CA, USA), or β-actin (internal control;1:500; Cell Signaling, MA, USA). After a 2h incubation with secondary antibodies (1:2,000; R&D, MN, USA) membranes were visualized using enhanced chemiluminescence reagents (Bio-Rad, CA, USA).

### Real-time polymerase chain reaction

Total RNA was extracted from BMSCs using the RNAeasy Mini kit (Qiagen, Germany) and reverse-transcribed to cDNA using the GoScript Reverse Transcription system (Promega, WI, USA). PCR primers, based on the NCBI nucleotide sequence database and designed using Primer Premier 5 software, were as follows: Nestin: Forward-tcc aga aac tcaa gca ccac-5′; Reverse: 5′-cca ccg tat ctt ccc acct-3′. β-actin: Forward: 5′-ggg aaa tcg tgc gtg acat-3′; Reverse: 5′-cag gag gag caa tga tctt-3′; PAX6: Forward: 5′-cag ctt ggt ggt gtc ttgt-3′; Reverse: 5′- ctt gga cgg gaa ctg acac-3. Oct4: Forward: 5′-gtg gag agc aac tcc gatg-3′; Reverse: 5′-tgc tcc agc ttc tcc ttc tc-3′. Each reaction contained 2 μl of cDNA, 3 μl of Green Premix and 2 μl of Nucleotidase-free H_2_O (both from Promega, WI, USA). PCR conditions were: 94°C and 55°C for 45s for denaturation and annealing, and 72°C for 1 min for extension; 50 cycles in total. PCR products were visualized using a Bio-Rad imaging system (BioRad, CA, USA).

### Statistical analysis

Statistical analyses were performed with SPSS v15.0 (IBM Software Inc.). P < 0.05 was considered statistically significant. *T-test* and One-way ANOVA were used for comparisons between different groups.
